# Guillain Barré syndrome associated with bladder instillation of Bacille Calmette Guérin (BCG)

**DOI:** 10.1099/jmmcr.0.005164

**Published:** 2018-08-20

**Authors:** Karmel Webb, Pradhib Venkatesan

**Affiliations:** Department of Infectious Diseases, Nottingham University Hospitals City Campus, Nottingham NG5 1PB, UK

**Keywords:** Bacille Calmette Guérin, BCG, disseminated BCGosis, Guillain Barré syndrome

## Abstract

**Introduction.:**

Guillain Barré Syndrome (GBS) has rarely been associated with tuberculosis and has been previously reported after Bacille Calmette Guérin (BCG) vaccination, but we report an association of GBS with intra-vesical BCG instillations followed by the clinical picture of disseminated BCGosis.

**Case presentation.:**

A 68-year-old man with bladder carcinoma had a transurethral tumour resection followed by repeated BCG instillations. Catheterization for his eighth dose was traumatic, causing frank haematuria. Ten days later he presented with fevers, myalgia and dyspnoea. Chest X-ray on admission showed micronodular shadowing and a computed tomography scan showed miliary changes in the lungs. Disseminated BCGosis infection was suspected and his symptoms did improve after starting rifamipicin, isoniazid and ethambutol. Over 2 weeks post-admission he developed an unsteady gait, reduced pin-prick sensation below both knees and fingertips, reduced proprioception in both toes and ankles, with absent reflexes in his lower limbs and diminished reflexes in his upper limbs. Nerve conduction studies showed a purely demyelinating sensori-motor peripheral neuropathy in upper and lower limbs, characteristic of GBS.

**Conclusion.:**

To our knowledge this is the first case report of GBS following bladder instillation of BCG. Given the millions of cases of tuberculosis and millions of doses of administered BCG, GBS must be a very rare adverse effect.

## Introduction

Guillain Barré syndrome (GBS) is usually preceded by an acute respiratory or gastrointestinal infection. Its association with tuberculosis has been reported in the literature, albeit rarely, and to our knowledge on only seven occasions [1–6]. GBS has been previously reported in association with Bacille Calmette Guérin (BCG) vaccination [7], but the present case report is the first to describe an association of GBS with intra-vesical BCG instillations, followed by the clinical picture of disseminated BCGosis.

## Case report

A 68-year-old -man with a history of coronary bypass grafts and stable Crohn’s disease, last treated with infliximab 11 months prior to presentation, was diagnosed with transitional cell carcinoma of the bladder and underwent a transurethral resection of the bladder tumour. He subsequently received six weekly intra-vesical instillations of BCG. He received maintenance therapy 3 months later, and on his second instillation for maintenance there was difficulty inserting a catheter and frank haematuria was noted following catheterization. After 11 days he presented with fevers of 39.1 °C, sweats, shivers, generalized myalgia and reduced appetite. On admission he also complained of shortness of breath on exertion, with no cough or sputum production initially, and right upper quadrant pain. On examination his chest was clinically clear and his oxygen saturation was 95 % on air. He had a palpable liver edge. A full neurological examination was normal. He was commenced on intravenous piperacillin/tazobactam.

**Fig. 1.**
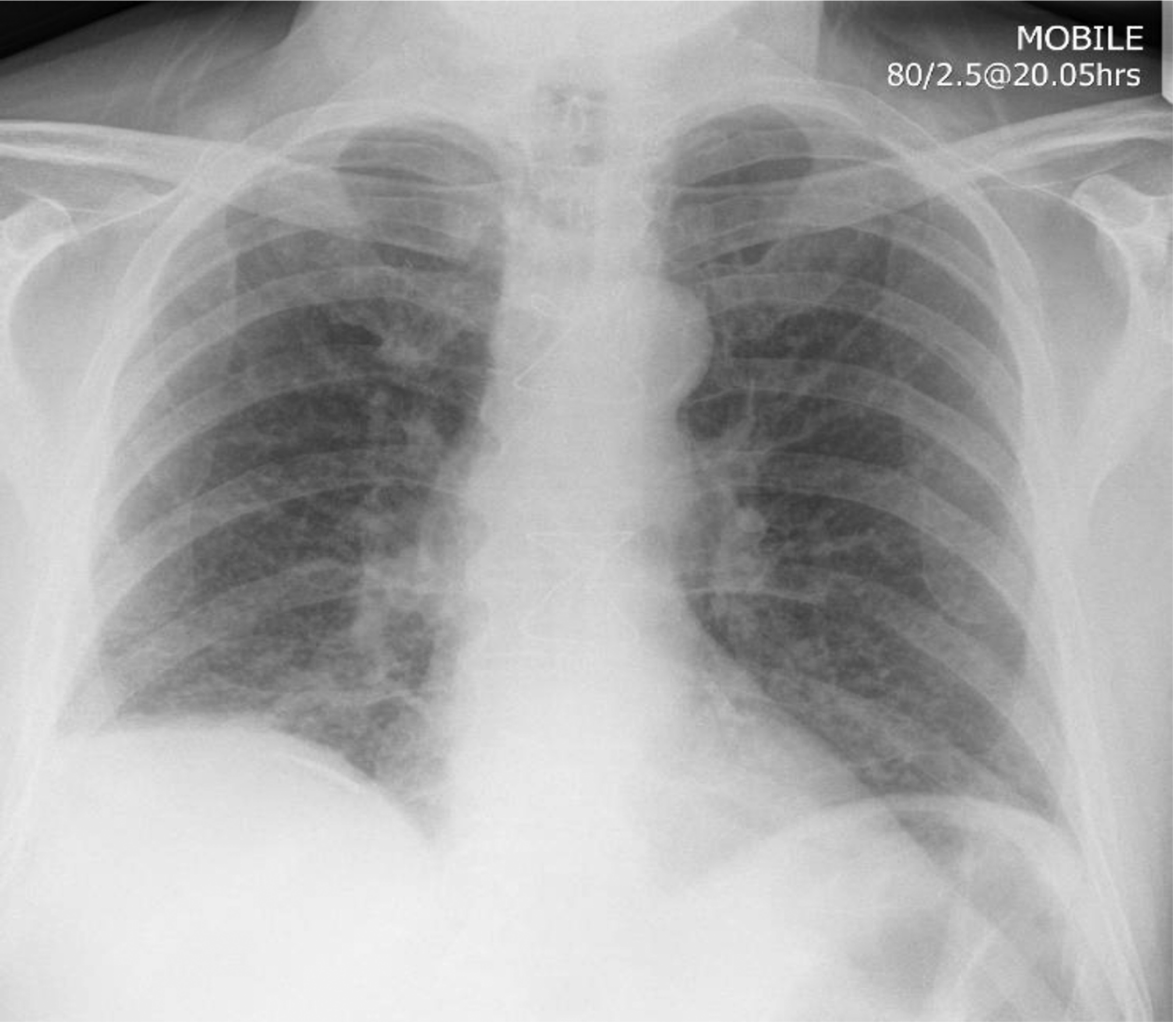
Anteroposterior erect chest X-ray showing fine micronodular shadowing.

**Fig. 2.**
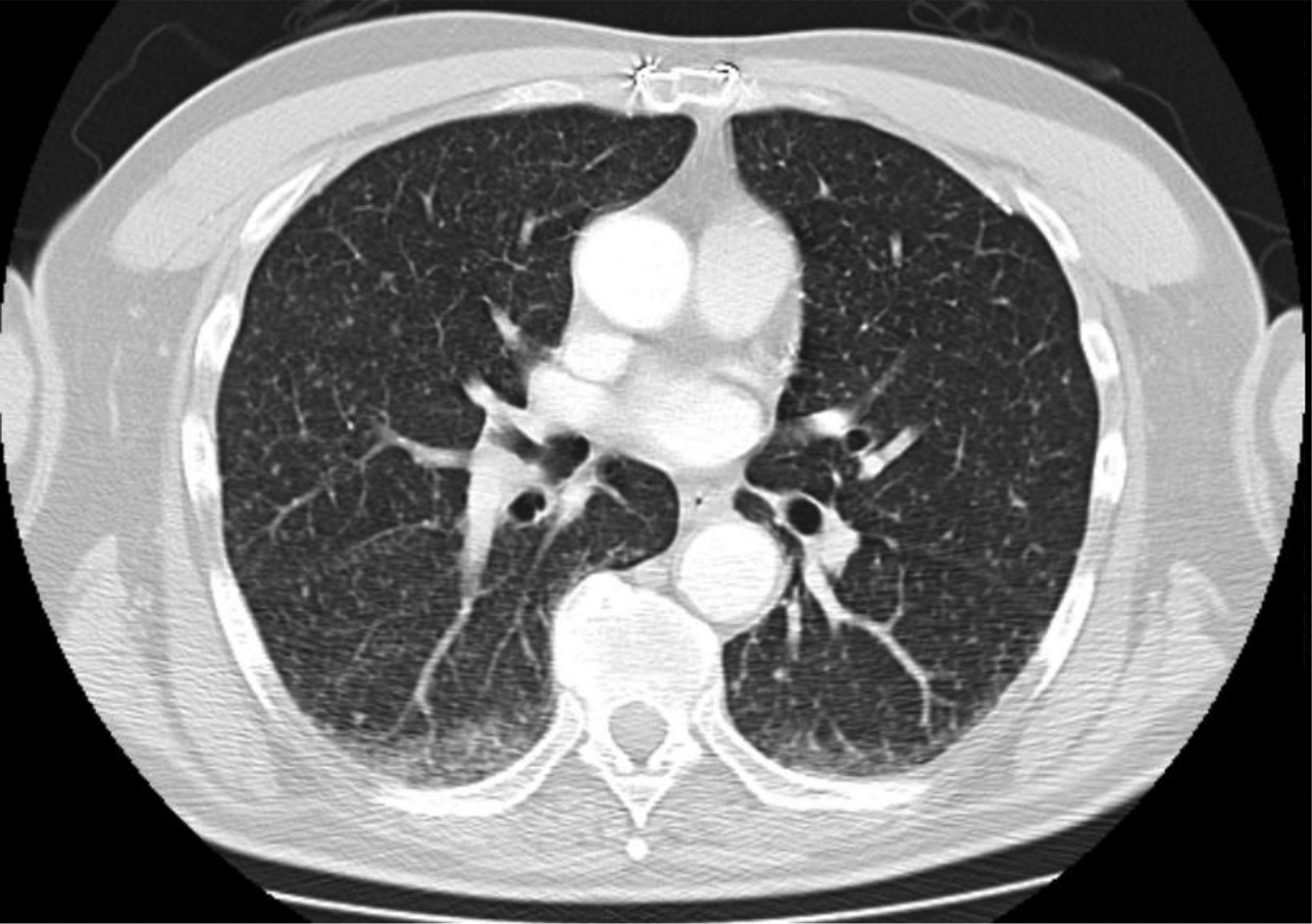
Computed tomography scan of chest with reported miliary changes.

Abnormal results on initial investigations included a thrombocytosis of 507×10^9^ l^−1^, alkaline phosphatase 150 U l^−1^ (normal range 40–130), an arterial blood gas with a *p*O_2_ of 10.2 kPa on air, C-reactive protein raised to 90 mg l^−1^ (normal <10) and a chest X-ray with micronodular shadowing in the mid- and lower zones bilaterally (Fig. 1). A subsequent computed tomography scan showed fine miliary shadows widespread in the lungs, consolidation at the right base and slight enlargement of the liver (Fig. 2).

There was no improvement with intravenous piperacillin/tazobactam and with the above results he was diagnosed clinically as having probable disseminated BCGosis infection. Subsequently he developed a productive cough and sputum. Blood and early morning urine samples were sent for mycobacterial cultures, prior to commencing rifampicin, isoniazid, ethambutol and pyridoxine 4 days after admission. On this treatment his temperature and cough settled. However, later all his mycobacterial cultures proved negative. Fourteen days after admission he began to develop an unsteady gait. On examination he had new reduced pin-prick sensation from the thigh downwards on both lower limbs and also in his fingertips. He had reduced proprioception in both toes and ankles. His reflexes were absent in his lower limbs and diminished in his upper limbs. He developed significant neuropathic pain in his legs which was relieved by amitryptilline. He also had postural hypotension with his systolic blood pressure falling from 128 mmHg lying to 105 mmHg on standing.

## Investigations

On investigating his postural hypotension two synacthen tests were normal. His vitamin B12 and folate levels were normal. Thyroid stimulating hormone was within normal limits. Anti-nuclear antibodies, dsDNA, ENA, cANCA, anti-PR3, anti-MPO, glycolipid antibodies, anti-MAG IgM, acetylcholine receptor antibodies, anti-Hu, anti-Yo, anti-Ri and anti-ampiphysin were all negative. There was no serum paraprotein present. Human immunodeficiency virus and syphilis serology were both negative. Ebstein Barr virus and cytomegalovirus (CMV) IgM were negative and CMV DNA was not detected. A magnetic resonance imaging (MRI) scan of the brain showed chronic small vessel disease. An MRI scan of the spine showed some disc degenerative changes but no significant nerve root impingement. The patient had a lumbar puncture with cerebrospinal fluid (CSF) protein elevated to 3305 mg l^−1^ (normal range 150–450). CSF glucose was 3.9 mmol l^−1^ compared with a concomitant serum glucose of 6.4 mmol l^−1^. In the CSF no white blood cells were seen nor organisms on microscopy with Gram or auramine stains. There was no subsequent growth after conventional cultures or for mycobacteria after 42 days of incubation. Nerve conduction studies showed a purely demyelinating type of sensory motor peripheral neuropathy in upper and lower limbs, when performed 7 days after the onset of neurological symptoms.

## Diagnosis

The clinical diagnosis of disseminated BCGosis was not fully confirmed by mycobacterial culture, but was compatible with the miliary changes noted after BCG bladder instillation. The traumatic nature of the catheterization would have injured the epithelium and facilitated the systemic entry of mycobacteria.

There was a clear peripheral neuropathy, without significant spinal cord or cerebral pathology, associated with raised protein in the CSF but no inflammatory cells. All of these findings were consistent with a diagnosis of GBS, in the absence of another explanation from a variety of investigations. The nerve conduction studies were reported as typical of GBS.

Usually GBS is preceded by a gastrointestinal infection, classically due to *Campylobacter*, or a respiratory tract infection [8]. CMV infection has been associated with GBS, but in this case there was no evidence for this. Various other infections have occasionally been associated with GBS. Apart from exposure to BCG we found no evidence of another pathogen that might have been a trigger.

Neurological features did develop 10 days after starting anti-mycobacterial treatment, accompanied by pyridoxine, but later his symptoms improved whilst remaining on this treatment, making the drugs themselves an unlikely contributor.

## Treatment

The patient was not commenced on any specific treatment for GBS, in the form of steroids or intravenous immunoglobulin.

## Outcome and follow-up

The patient was discharged once his fever, cough and breathlessness had improved. One month after discharge, it was noted that whilst tone and power of all four limbs were normal, there was still some residual blunting of pin-prick sensation in both feet, mostly on the lateral aspect of the right leg. Reflexes were restored and normal, except for an equivocal plantar reflex on the right. By 6 months sensation was subjectively normal and there were no residual neurological features.

## Discussion

GBS is an acquired inflammatory peripheral neuropathy defined by acute onset, raised CSF protein and a monophasic course with partial or total recovery [8]. Immune mechanisms are believed to be responsible for neuropathy, rather than pathogens directly causing perineural infection, therefore explaining improvements with immunomodifying treatments. Immune mechanisms may depend on molecular mimicry between epitopes present in pathogens cross-reacting with host targets. The requirement for molecular mimicry may limit the number of pathogens capable of triggering GBS. Epidemiological evidence is strongest for *Campylobacter* and CMV. In 40 % of cases, no cause or trigger is found.

To our knowledge, there have been seven published cases of GBS associated with *Mycobacterium tuberculosis* infection [1–6]. In addition there is one case report of a chronic inflammatory demyelinating polyneuropathy associated with intestinal tuberculosis [9]. In addition it is recognized that spinal infection can lead to direct involvement of peripheral nerve roots.

BCG is derived from *Mycobacterium bovis*, and with repeated passage was attenuated with loss of the RD1 section of its genome. This work was undertaken by Albert Calmette and Camille Guérin. They found that culturing mycobacteria in ox bile, glycerine and potato medium prevented clumping but also attenuated the organism. After 3 weekly subcultures between 1902 and 1919 they derived a live strain incapable of causing disease in various animal hosts. In 1921 its first human administration was to a newborn infant. We have found one case report of GBS after BCG vaccination in an adolescent girl [7]. Intra-vesical BCG post-marketing surveillance records nervous system disorders with an incidence of <1 : 10 000. These side-effects include complaints of dizziness, dysaesthesia, hyperaesthesia, paraesthesia, somnolence, headache and neuralgia, but not specifically the syndrome of GBS [10]. To our knowledge this is the first case report of GBS following bladder instillation of BCG. Given the millions of cases of tuberculosis and millions of doses of administered BCG, GBS must be a very rare adverse effect.
